# Care seek behavior for low back pain in southern Brazil during the COVID-19 pandemic: a panel data analysis

**DOI:** 10.1186/s12891-023-06538-z

**Published:** 2023-06-07

**Authors:** Eduardo L Caputo, Natan Feter, Ricardo N C Pinto, Felipe Mendes Delpino, Júlia Cassuriaga, Carine N da Silva, Jayne S Leite, Jeferson S Jerônimo, Sophia D P da Silva, Felipe F Reichert, Airton J Rombaldi, Marcelo C da Silva

**Affiliations:** 1 Programa de Pós-Graduação em Educação Física, Universidade Federal de Pelotas, Pelotas, Brazil; 2 School of Physical Education, Federal University of Pelotas, Luís de Camões Street, 625, Pelotas-RS, Pelotas, 96055-630 Brazil; 3 Programa de Pós-Graduação em Epidemiologia, Universidade Federal do Rio Grande do Sul, Rio Grande do Sul, Brazil; 4 Programa de Pós-Graduação em Enfermagem, Universidade Federal de Pelotas, Pelotas, Brazil; 5 Programa de Pós-Graduação em Ciências da Saúde, Universidade Federal do Rio Grande, Rio Grande, Brazil; 6 Programa de Pós-Graduação em Ciências da Saúde, Universidade Federal do Rio Grande do Sul, Porto Alegre, Brazil

**Keywords:** Low back pain, COVID-19, Care seek

## Abstract

**Background:**

During the COVID-19 pandemic, people with low back pain (LBP) might have avoided seeking care for their pain. We aimed to investigate how the COVID-19 pandemic has affected LBP care seeking behavior among adults.

**Methods:**

Data from four assessments of the PAMPA cohort were analyzed. Participants who reported experiencing LBP during wave one both before and during social restrictions (n = 1,753 and n = 1,712, respectively), wave two (n = 2,009), and wave three (n = 2,482) were included. We asked participants about sociodemographic, behavioral, and health factors and outcomes related to LBP. Poisson regression analyses were conducted, and data are presented as prevalence ratios (PR) and respective 95% confidence interval (95%CI).

**Results:**

Overall, care seeking behavior decreased by half in the first months of restrictions, from 51.5% to 25.2%. Although there was an increase in care seeking behavior observed in the other two assessments (nearly 10 and 16 months after restrictions), it was insufficient to reach pre-pandemic levels. In the first months of restrictions, a similar scenario was observed for specific care, such as general practitioner and exercise professional care, with proportions of pre-pandemic levels reached after 10 and 16 months. Women were more likely to seek care for LBP 10 and 16 months after restrictions (PR 1.30 95%CI 1.11; 1.52, PR 1.22 95%CI 1.06; 1.39, respectively). Also, those participants who worked, were physically active, and reported pain-related disability and high pain levels were more likely to seek care at all time points assessed.

**Conclusion:**

Overall, care-seeking behavior for LBP significantly decreased in the first months of restrictions and increased in the following months; however, this behavior remained lower than pre-pandemic levels.

**Supplementary Information:**

The online version contains supplementary material available at 10.1186/s12891-023-06538-z.

## Background

Low back pain (LBP) is a public health concern due to its high incidence rates and associated disability leading to work absences, adversely affecting social and economic factors [1]. LBP incidence may be directly related to inappropriate postures while working, extended periods of sitting, physical inactivity, and high stress and anxiety levels [1, 2]. During COVID-19 the pandemic restrictions, a decrease in physical activity levels and an increase in anxiety and depression was observed, being strongly related to LBP outcomes in the initial months of the restrictions [3–5].

The COVID-19 pandemic has impaired healthcare access, as health services have been reorganized to comply with pandemic demands [6]. Inadequate pain management can worsen patients health conditions, reducing the quality of life and causing workplace absenteeism, sleep disturbances, and psychological and social issues [7]. Despite concerns about LBP care by health centers, especially regarding acute cases, it is reasonable to hypothesize that people experiencing LBP may have avoided seeking care due to the restrictive measures implemented during the pandemic [8].

A systematic review indicated that the pooled prevalence of care seeking for LBP in high-income countries (e.g., the UK, USA, and Australia) was 56% [9]. Sociodemographic factors (e.g., sex and age), pain-related characteristics (e.g., intensity and disability), employment status, and beliefs about the pain were associated with increased care seeking for LBP before the pandemic [9]. Thus, we aimed to investigate how the COVID-19 pandemic has affected LBP care seeking among adults living in the southernmost state of Brazil.

## Methods

### Study design and participants

We conducted a panel analysis using the Prospective Study about Mental and Physical Health (PAMPA) Cohort data. Participants were adults (18 or older) living in the Rio Grande do Sul state, Brazil. The institutional ethics board approved the study (protocol: 4.093.170). Details regarding the study design and methodology can be found elsewhere [10].

Participants were recruited using social media campaigns and spreading the questionnaire link through researchers’ personal and professional contacts (i.e., snowball strategy). Data assessment took place in June/July 2020 (wave one, retrospectively, and cross-sectionally), December 2020/January 2021 (wave two), and June/July 2021 (wave three). Waves one, two, and three were conducted after nearly three, 10, and 16 months of social restrictions were implemented in the Rio Grande do Sul state, respectively. All data were assessed using a self-reported, self-administered electronic questionnaire developed in Google forms (wave one) and RedCap (waves two and three) [11]. The sample for this study consisted of participants who reported experiencing LBP during wave one before and during the social restrictions (n = 1,753 and n = 1,712, respectively), wave two (n = 2,009), and wave three (n = 2,482).

### LBP intensity, activity limitation, and care seeking

Participants were asked about LBP experience at all time points. In wave 1, participants were asked about LBP variables retrospectively (i.e., before social restrictions) and during social restrictions (i.e., first three months); in waves two and three, LBP variables referred to the last six months. Those who answered “yes” to LBP experience were asked about pain intensity, activity limitation, and care seeking. A numeric pain rating scale was used to assess pain intensity, ranging from 0 (no pain) to 10 (worse pain). Activity limitation was assessed by asking participants if their pain was strong enough to limit or change their daily activities for at least one day [5].

Care seeking for LBP was assessed through the following question: *“Have you sought the following health professionals to treat your low back pain? (General practitioner, physiotherapist, exercise professional, other, and no/none)”*. Based on this question, we built a binary variable for care seeking behavior (yes or no) and four variables regarding each healthcare professional.

### Covariates

Sociodemographic (sex, age, ethnicity, and marital status), economic (decreased monthly income), behavioral (physical activity), health (chronic diseases and symptoms of anxiety and depression), and work status were used as covariates in adjusted analyses [10]. Physical activity was assessed by days per week and minutes per day of activities performed on the leisure time in the last seven days. Those who engaged in < 150 min/week were defined as inactive, and those who engaged in ≥ 150 min/week were defined as physically active [12]. Diagnosis of chronic diseases was assessed using a question of the Brazilian System of Surveillance of Risk Factors for Chronic Diseases by Telephone Interview – VIGITEL [13]. The Hospital Anxiety and Depression Scale (HADS) was used to assess symptoms of anxiety and depression in the last weeks and before the pandemic. This scale has 14 items, seven in each domain (i.e., depression and anxiety). The maximum score is 21 points, as each item can be scored from 0 to 3. Thus, participants were classified based on their scores as follows: non-cases (less than 7 points), mild cases (between 8 and 10), moderate cases (between 11 and 14), and severe cases (between 15 and 21).

### Data analyses

Descriptive data are presented as means or proportions, followed by their respective 95% confidence interval (95%CI). The variation of care seeking proportions among waves was analyzed using a variance-weighed least squares regression, and estimates are presented as percentage points (pp). A p-value > 0.05 indicates stability, and a p < 0.05 indicates increasing or decreasing estimates. Poisson regression analyses assessed the association between outcome (care seek) and covariates. We included all variables in adjusted analyses regardless of the p-value in crude analyses. Data from regression analyses are presented as prevalence ratio (PR) and their respective 95%CI. We performed weighting procedures for state regions (*Sul, Norte, Missioneira, Metropolitana, Serra, Centro-Oeste, and Vales*, names in Portuguese) in all analyses since one region (Sul) has more respondents than others. Stata 15.1 was used, and a p-value of < 0.05 was considered for statistical significance.

## Results

Most participants who reported seeking care for LBP were women, under 60, white, and living with a partner. More than half of the participants who sought care reported activity limitation related to LBP after 10 and 16 months of restrictions, and pain intensity ranged from 6.0 to 6.4 among waves. The complete characteristics of participants who sought care for LBP are displayed in Supplementary Material 1.

**Fig. 1:**
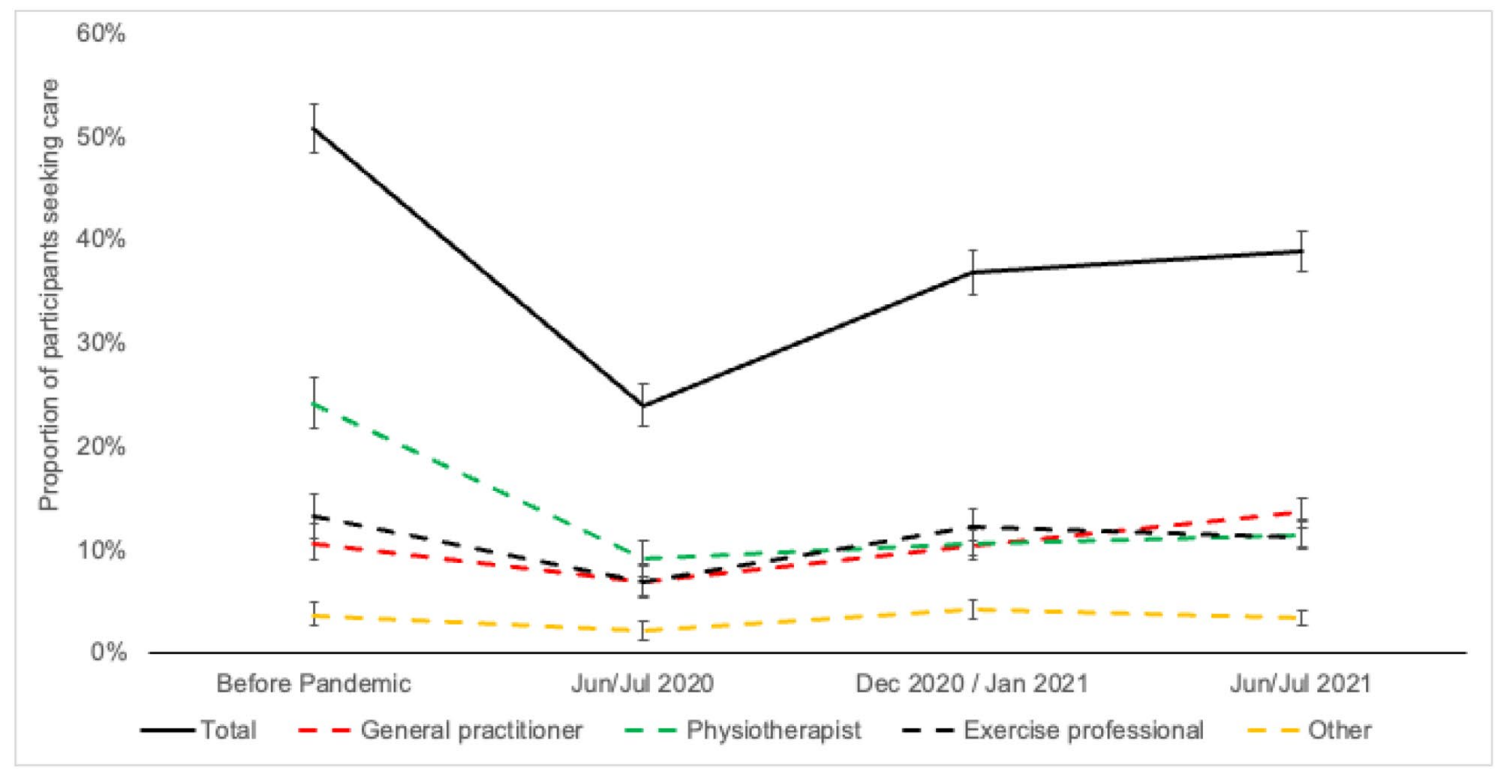
Prevalence of care seek behavior for LBP in the four data assessments by type of health care professional. Data are presented as proportions and their respective 95%CI. Rio Grande do Sul, Brazil

The proportion of care seeking behavior among waves is presented in Fig. 1. Overall, the proportion of participants who sought care decreased in the first months of restrictions for the total sample (51.5% to 25.2%), physiotherapist (PT) (24.1% to 9.1%), general practitioner (GP) (10.7% to 6.9%) and exercise professional (13.2% to 7.0%). A negative trend was observed for the total sample (-1.3 pp; p = 0.009) and PT (-2.3 pp; p < 0.001), with proportions remaining reduced over time when compared to before the pandemic. On the other hand, GP and exercise professionals showed positive trends (1.0 pp; p = 0.001 and 0.7 pp; p = 0.035. respectively) and reached pre-pandemic levels over time.

The crude analyses between care seek and covariates can be found in Supplementary Material 2. The adjusted analyzes are displayed in Table 1. Female sex was associated with increased care seek 10 and 16 months after restrictions (PR 1.30 95%CI 1.11; 1.52, PR 1.22 95%CI 1.06; 1.39, respectively). Being 60 years old or over and having a chronic disease was not associated with care seek only in the first three months of restrictions. Moderate symptoms of depression decreased the likelihood of seeking care for LBP in all time points (PR 0.68 [95%CI 0.52; 0.90], PR 0.72 [95%CI 0.61; 0.86], PR 0.80 [95%CI 0.70; 0.92]). Participants who worked, were physically active, and reported disability and increased pain intensity were more likely to seek care for their pain at all time points assessed.

**Table 1:** Factors associated with care seeking behavior for low back pain. PAMPA cohort

	Before Pandemic	Jun/Jul 2020	Dec 2020/Jan 2021	Jun/Jul 2021
*Sex*				
Male	1.00	1.00	1.00	1.00
Female	1.04 (0.90; 1.19)	1.10 (0.85; 1.43)	**1.30 (1.11; 1.52)**	**1.22 (1.06; 1.39)**
*Age (years)*				
18–30	1.00	1.00	1.00	1.00
31–59	**1.18 (1.03; 1.34)**	1. 08 (0.84; 1.39)	1.02 (0.88; 1.18)	1.04 (0.92; 1.17)
60+	**1.32 (1.10; 1.58)**	1.12 (0.76; 1.67)	**1.58 (1.29; 1.93)**	**1.24 (1.03; 1.48)**
*Ethnicity*				
White	1.00	1.00	1.00	1.00
Mixed	0.99 (0.84; 1.19)	1.15 (0.83; 1.59)	0.94 (0.77; 1.14)	0.81 (0.68; 0.97)
*Conjugal status*				)
With partner	1.00	1.00	1.00	1.00
Without partner	0.98 (0.87; 1.10)	**0.70 (0.56; 0.88)**	0.99 (0.88; 1.13)	1.04 (0.94; 1.15)
*Work status*	^1^	^1^		
No			1.00	1.00
Yes			**1.28 (1.11; 1.47)**	**1.25 (1.12; 1.40)**
*Monthly income reduced since COVID-19*	^1^			
No		1.00	1.00	1.00
Yes		1.11 (0.91; 1.35)	**1.22 (1.02; 1.46)**	1.14 (0.95; 1.36)
*Physical activity*				
Inactive	1.00	1.00	1.00	1.00
Active	**1.19 (1.07; 1.33)**	**1.75 (1.41; 2.16)**	**1.69 (1.51; 1.90)**	**1.74 (1.58; 1.93)**
*Chronic disease*				
No	1.00	1.00	1.00	1.00
Yes	**1.21 (1.07; 1.39)**	1.21 (0.97; 1.51)	**1.18 (1.01; 1.37)**	**1.30 (1.13; 1.49)**
*Depression symptoms*				
Normal	1.00	1.00	1.00	1.00
Mild	0.84 (0.71; 1.00)	1.03 (0.82; 1.29)	**0.84 (0.74; 0.95)**	0.90 (0.81; 1.01)
Moderate	0.94 (0.71; 1.25)	**0.68 (0.52; 0.90)**	**0.72 (0.61; 0.86)**	**0.80 (0.70; 0.92)**
Severe	0.74 (0.22; 2.36)	**0.45 (0.24; 0.87)**	0.76 (0.49; 1.16)	0.73 (0.49; 1.10)
*Anxiety symptoms*				
Normal	1.00	1.00	1.00	1.00
Mild	**0.85 (0.74; 0.97)**	1.05 (0.80; 1.38)	1.01 (0.87; 1.16)	1.07 (0.94; 1.22)
Moderate	0.83 (0.63; 1.10)	0.99 (0.73; 1.35)	0.96 (0.81; 1.15)	0.97 (0.84; 1.13)
Severe	^2^	1.16 (0.82; 1.66)	0.73 (0.52; 1.03)	1.03 (0.83; 1.29)
*Disability*				
No	1.00	1.00	1.00	1.00
Yes	**1.34 (1.16; 1.54)**	**1.40 (1.09; 1.80)**	**1.30 (1.11; 1.52)**	**1.38 (1.22; 1.57)**
*Pain intensity*	**1.10 (1.07; 1.14)**	**1.12 (1.05; 1.18)**	**1.10 (1.06; 1.14)**	**1.10 (1.07; 1.14)**

Results were calculated using Poisson regression models with robust variance simultaneously adjusted for all the covariates in the survey period. Data are presented as PR (95%CI).

Bold numbers indicate statistical significance (p < 0.05)

^1^ Variable not assessed in the specific survey

^2^ Insufficient number of participants in this category in the specific survey

PR = prevalence ratio; CI = confidence interval

## Discussion

We revealed that care seeking for LBP decreased in the first months of restrictions and remained at reduced levels after nearly 16 months. PT was the healthcare professional most sought before the pandemic; however, the proportions remained reduced and similar to GP and exercise professionals during restrictions. Those who reported symptoms of depression were less likely to seek care for their pain during restrictions. On the other hand, women aged 60 or over, physically active, and working participants were more likely to seek care. Also, those who reported activity limitation related to and increased pain intensity were more likely to seek care for their pain.

As previously stated, the pooled prevalence of LBP care seeking behavior in high-income countries was 56% [9], which is similar to the prevalence reported before restrictions (51.5%). While data on LBP seek for low- and medium-income countries are still lacking, we provide evidence that LBP care was impaired during COVID-19 restrictions in Southern Brazil. It is well recognized that the pandemic stroked healthcare systems, with more people infected and requiring health assistance [14]. Consequently, care seeking for LBP was expected to decrease in the first months of restrictions. However, we should highlight that vaccination was already started in the last assessment (wave three), and restrictions were eased in Brazil. Nevertheless, our data suggests that people with LBP still feel uneasy about seeking care.

Previous evidence showed that GP is highly sought for people with LBP, followed by PT [15, 16]. Specialized services such as PT are usually sought through a GP referral in the Brazilian public health system (the Unified Health System (SUS, in Portuguese)). However, SUS is the leading healthcare source for the low-income population [17]. Since most of our study sample is highly educated and consequently from mid- or high-income [10] and, in the private system, people are allowed to seek PT without a GP referral, this might explain the high proportion of participants who sought a PT before the pandemic. Also, although exercise interventions are one of the main strategies recommended to prevent and manage LBP, exercise professionals are usually not reported in the literature [9]. These professionals can help LBP patients by promoting exercises and supporting them to keep a healthy lifestyle.

The relationship between the female sex and care-seeking for LBP remains inconsistent in the literature. Most studies did not report a significant association between care seek and the female sex [9]. This corroborates our findings since we only found significant associations in two assessments (i.e., 10 and 16 months after restrictions). Regarding age, our data confirms studies conducted before the pandemic, which showed a relationship between age and care seek [18, 19]. Older individuals are considered a risky COVID-19 population, and the increased concern about COVID-19 safety measures on this population might explain the lack of association in the first months of restrictions.

Unlike previous studies from high-income countries, we showed a significant association between working and LBP care seek [9]. As previously stated, this might be explained by the lack of care seeking data in low- and middle-income countries [9]. In the last years, the exposure to risk factors associated with LBP disability (e.g., psychosocial factors and work organization support) increased, and COVID-19 might worsen this scenario due to its economic impact [20]. In addition, the pandemic-related changes in peoples routines when working from home, such as long working hours, uncomfortable furniture, and inappropriate postures, should be considered [21]. Strategies to stimulate multifaceted workplace interventions (e.g., cognitive behavioral therapy and developing return-to-work plans) can help decrease the LBP burden in healthcare systems [22].

Even though LBP care seek is associated with other variables (e.g., sociodemographic), others have reported that pain-related characteristics, such as intensity and disability, are strictly related to seeking care [18, 19, 23, 24]. We previously showed that people with chronic conditions avoid seeking care during the first months of restrictions [25]. The association between pain characteristics and maintenance sought remained even when restrictions were strengthened (nearly the first three and ten months), and COVID-19 cases increased sharply. Therefore, it is likely that people with LBP sought care regardless of the pandemic situation due to the limitations that high pain intensity imposed on their daily life activities.

The limitations of our study should be acknowledged. First, since data collection procedures were online, and many people do not have internet access in Brazil, an overrepresentation of some subgroups was observed in our sample (i.e., younger and more schooled individuals were overrepresented compared to the Rio Grande do Sul state population) [26]. Second, we were only able to assess work status in waves two and three, not allowing any inferences on the short-term effects of this variable on care-seek behavior. Third, recall bias might be expected because the first wave had a retrospective characteristic, and no specific time point was established regarding the pre-pandemic period. However, we provided data for a critical LBP-related outcome lacking in Brazil and other middle-income countries. We could help stakeholders and policymakers develop strategies to decrease the LBP burden.

## Conclusion

People experiencing LBP during pandemic restrictions initially avoided seeking care for their pain, which significantly decreased in the first months of restrictions in Rio Grande do Sul state, remaining reduced over time compared to pre-pandemic levels. GP and exercise professionals were highly sought nearly after 10 and 16 months of restrictions besides PT. Finally, sociodemographic, work, health, and pain characteristics were strictly related to this behavior. Further studies should focus on specific populations and the reasons influencing people to seek care from different health professionals.

## Electronic supplementary material

Below is the link to the electronic supplementary material.

Supplementary Material 1

Supplementary Material 2

## Data Availability

The datasets used and/or analyzed during the current study are available from the corresponding author on reasonable request.
