# Supplementary material for: Care seek behavior for low back pain in southern Brazil during the COVID-19 pandemic: a panel data analysis
Source: BMC Musculoskelet Disord. 2023 Jun 7;24:466. doi: 10.1186/s12891-023-06538-z (PMC10244084; doi:10.1186/s12891-023-06538-z)
Supplement: Supplementary file 1 — Supplementary Material 1 [file 12891_2023_6538_MOESM1_ESM.docx]

| **Supplementary Material 1.** Sociodemographic, economic, behavioral and health characteristics of participants who sought care for low back pain. Rio Grande do Sul, Brazil. Data are presented as proportions with their respective 95%CI. | | | | |
| --- | --- | --- | --- | --- |
|  | **Before pandemic**  **(n=897)** | **Jun/Jul 2020**  **(n=419)** | **Dec 2020/Jan 2021**  **(n=739)** | **Jun/Jul 2021**  **(n=978)** |
| *Sex* |  |  |  |  |
| Male | 19.9 (16.8; 23.4) | 17.6 (13.5; 22.6) | 17.9 (15.3; 21.0) | 16.4 (14.1; 18.9) |
| Female | 80.1 (76.6; 83.2) | 82.4 (77.4; 86.5) | 82.0 (78.9; 84.7) | 83.6 (81.0; 85.9) |
| *Age (years)* |  |  |  |  |
| 18-30 | 30.1 (26.4; 34.0) | 28.5 (23.4; 34.4) | 25.8 (22.6; 29.2) | 26.1 (23.3; 29.2) |
| 31-59 | 59.4 (55.3; 63.4) | 62.2 (56.2; 67.9) | 62.4 (58.6; 65.9) | 63.2 (59.9; 66.4) |
| 60+ | 10.5 (8.2; 13.3) | 9.2 (6.4; 12.9) | 11.9 (9.6; 14.6) | 10.6 (8.7; 12.9) |
| *Ethnicity* |  |  |  |  |
| White | 91.7 (8.9; 9.4) | 91.4 (87.4; 94.2) | 90.1 (87.7; 92.1) | 91.1 (89.1; 92.8) |
| Mixed | 8.3 (6.3; 10.9) | 8.6 (5.8; 12.6) | 9.9 (7.9; 12.3) | 8.9 (7.2; 10.9) |
| *Marital status* |  |  |  |  |
| With partner | 65.3 (61.3; 69.1) | 71.0 (65.3; 76.0) | 63.2 (59.5; 66.7) | 65.8 (62.6; 68.8) |
| Without partner | 34.7 (30.9; 38.7) | 29.0 (23.9; 34.7) | 36.8 (33.3; 40.5) | 34.2 (31.1; 37.4) |
| *Work status* |  |  |  |  |
| No | - | - | 22.9 (19.9; 26.3) | 25.4 (22.7; 28.4) |
| Yes | - | - | 77.0 (73.7; 80.0) | 74.6 (71.6; 77.3) |
| *Monthly income reduced since COVID-19* |  |  |  |  |
| No | - | 49.8 (43.8; 55.8) | 89.0 (86.4; 91.2) | 92.4 (90.5; 93.9) |
| Yes | - | 50.2 (44.2; 56.2) | 11.0 (8.8; 13.6) | 7.6 (6.0; 9.5) |
| *Physical activity* |  |  |  |  |
| Inactive | 59.3 (55.2; 63.3) | 68.1 (62.2; 73.4) | 59.5 (55.7; 63.1) | 61.2 (57.9; 64.4) |
| Active | 40.7 (36.7; 44.8) | 31.9 (26.6; 37.8) | 40.5 (37.9; 44.3) | 38.8 (35.6; 42.1) |
| *Chronic disease* |  |  |  |  |
| No | 30.7 (27.0; 34.7) | 32.8 (27.3; 38.7) | 20.3 (17.5; 23.5) | 18.6 (16.1; 21.4) |
| Yes | 69.3 (65.4; 72.9) | 67.2 (61.3; 72.7) | 79.7 (76.5; 82.5) | 81.4 (78.6; 83.8) |
| *Depression symptoms* |  |  |  |  |
| Normal | 83.1 (79.7; 86.0) | 47.2 (41.3; 53.3) | 50.5 (46.8; 54.2) | 45.9 (42.7; 49.2) |
| Mild | 12.3 (9.7; 15.3) | 30.1 (24.9; 35.9) | 31.9 (28.5; 35.4) | 31.9 (28.9; 35.1) |
| Moderate | 4.4 (2.9; 4.5) | 19.6 (15.3; 24.8) | 15.8 (13.2; 18.6) | 20.2 (17.7; 23.0) |
| Severe | 0.3 (0.1; 1.3) | 3.1 (1.6; 5.9) | 1.8 (1.1; 3.2) | 1.8 (1.1; 2.9) |
| *Anxiety symptoms* |  |  |  |  |
| Normal | 75.4 (71.8; 78.7) | 37.9 (32.2; 43.9) | 30.0 (26.7; 33.5) | 28.4 (25.5; 31.5) |
| Mild | 20.5 (17.5; 23.9) | 23.4 (18.6; 28.9) | 37.5 (33.9; 41.2) | 34.1 (31.0; 37.3) |
| Moderate | 4.1 (2.8; 5.9) | 21.8 (17.3; 27.2) | 27.9 (24.7; 31.4) | 29.0 (26.1; 32.1) |
| Severe | - | 16.9 (12.9; 21.7) | 4.5 (3.2; 6.2) | 8.4 (6.8; 10.5) |
| *Disability* |  |  |  |  |
| No | 55.2 (51.1; 59.3) | 51.5 (45.5; 57.4) | 48.3 (44.6; 52.1) | 45.8 (42.5; 49.1) |
| Yes | 44.8 (40.7; 48.9) | 48.5 (42.6; 54.5) | 51.7(47.9; 55.4) | 54.2 (50.9; 57.5) |
| *Pain intensity** | 6.0 (5.8; 6.2) | 6.4 (6.1; 6.7) | 6.2 (6.0; 6.3) | 6.2 (6.1; 6.4) |

*Mean
