# Supplementary material for: Care seek behavior for low back pain in southern Brazil during the COVID-19 pandemic: a panel data analysis
Source: BMC Musculoskelet Disord. 2023 Jun 7;24:466. doi: 10.1186/s12891-023-06538-z (PMC10244084; doi:10.1186/s12891-023-06538-z)
Supplement: Supplementary file 2 — Supplementary Material 2 [file 12891_2023_6538_MOESM2_ESM.docx]

| **Supplementary Material 2.** Crude poison regression analyzes for LBP care seeking behavior. Data are presented as PR (95%CI). Rio Grande do Sul, Brazil. | | | | |
| --- | --- | --- | --- | --- |
|  | **Before Pandemic** | **Jun/Jul 2020** | **Dec 2020/Jan 2021** | **Jun/Jul 2021** |
| Sex |  |  |  |  |
| Male | 1.00 | 1.00 | 1.00 | 1.00 |
| Female | 1.08 (0.93; 1.24) | 1.19 (0.90; 1.57) | **1.23 (1.05; 1.44)** | **1.20 (1.04; 1.38)** |
| *Age (years)* |  |  |  |  |
| 18-30 | 1.00 | 1.00 | 1.00 | 1.00 |
| 31-59 | **1.33 (1.16; 1.52)** | **1.41 (1.11; 1.80)** | 1.13 (0.98; 1.30) | **1.17 (1.04; 1.32)** |
| 60+ | **1.59 (1.32; 1.91)** | 1.44 (0.98; 2.10) | **1.61 (1.32; 1.95)** | **1.28 (1.07; 1.54)** |
| *Ethnicity* |  |  |  |  |
| White | 1.00 | 1.00 | 1.00 | 1.00 |
| Mixed | 0.98 (0.80; 1.21) | 1.04 (0.73; 1.50) | 0.92 (0.76; 1.12) | 0.82 (0.68; 0.99) |
| *Marital status* |  |  |  |  |
| With partner | 1.00 | 1.00 | 1.00 | 1.00 |
| Without partner | **0.86 (0.76; 0.98)** | **0.67 (0.54; 0.85)** | 1.01 (0.89; 1.13) | 1.04 (0.93; 1.16) |
| *Work status* |  |  |  |  |
| No | - | - | 1.00 | 1.00 |
| Yes | - | - | **1.32 (1.14; 1.53)** | **1.22 (1.08; 1.38)** |
| *Monthly income reduced since COVID-19* |  |  |  |  |
| No | - | 1.00 | 1.00 | 1.00 |
| Yes | - | 1.12 (0.91; 1.37) | **1.20 (1.01; 1.43)** | 1.07 (0.89; 1.29) |
| *Physical activity* |  |  |  |  |
| Inactive | 1.00 | 1.00 | 1.00 | 1.00 |
| Active | 1.08 (0.96; 1.21) | **1.64 (1.33; 2.04)** | **1.48 (1.32; 1.67)** | **1.57 (1.42; 1.74)** |
| *Chronic disease* |  |  |  |  |
| No | 1.00 | 1.00 | 1.00 | 1.00 |
| Yes | **1.39 (1.23; 1.59)** | **1.38 (1.10; 1.73)** | **1.32 (1.13; 1.54)** | **1.41 (1.23; 1.63)** |
| *Depression symptoms* |  |  |  |  |
| Normal | 1.00 | 1.00 | 1.00 | 1.00 |
| Mild | 0.89 (0.74; 1.07) | 1.06 (0.84; 1.35) | 0.90 (0.79; 1.03) | 0.93 (0.83; 1.05) |
| Moderate | 0.95 (0.71; 1.26) | **0.70 (0.53; 0.93)** | **0.73 (0.62; 0.87)** | **0.83 (0.72; 0.95)** |
| Severe | 0.71 (0.18; 2.77) | **0.28 (0.24; 0.32)** | 0.76 (0.48; 1.18) | 0.85 (0.57; 1.25) |
| *Anxiety symptoms* |  |  |  |  |
| Normal | 1.00 | 1.00 | 1.00 | 1.00 |
| Mild | 0.90 (0.78; 1.04) | 1.04 (0.79; 1.37) | 1.03 (0.89; 1.18) | 1.09 (0.96; 1.24) |
| Moderate | 0.82 (0.60; 1.12) | 0.84 (0.63; 1.11) | 0.94 (0.80; 1.09) | 0.97 (0.85; 1.11) |
| Severe | - |  | **0.70 (0.52; 0.95)** | 1.08 (0.89; 1.32) |
| *Disability* |  |  |  |  |
| No | 1.00 | 1.00 | 1.00 | 1.00 |
| Yes | **1.86 (1.67; 2.06)** | **1.82 (1.48; 2.22)** | **1.64 (1.46; 1.84)** | **1.72 (1.56; 1.91)** |
| *Pain intensity* | **1.15 (1.13; 1.18)** | **1.15 (1.10; 1.21)** | **1.14 (1.11; 1.17)** | **1.14 (1.11; 1.17)** |

*PR = prevalence ratio

CI = confidence interval
